# Development of DNA Melt Curve Analysis for the Identification of Lepidopteran Pests in Almonds and Pistachios

**DOI:** 10.3390/insects12060553

**Published:** 2021-06-15

**Authors:** Rohith Vulchi, Kent M. Daane, Jacob A. Wenger

**Affiliations:** 1 Department of Plant Science, California State University, Fresno, 2415 E. San Ramon Ave, Fresno, CA 93740-8033, USA; vulc18@tamu.edu; 2 Department of Environmental Science, Policy, & Management, University of California Berkeley, Berkeley, CA 94720-3114, USA; kmdaane@berkeley.edu

**Keywords:** quantitative PCR, real-time PCR, pest identification, orchards, diagnostic protocol, melt curve profile, universal primers

## Abstract

**Simple Summary:**

Almonds and pistachios are fed upon by a diverse assemblage of lepidopteran insects, several of which are economically important pests. Unfortunately, identification of these pests can be difficult, as specimens are frequently damaged during collection, occur in traps with non-target species, and are morphologically similar up to their third instar. Here, we present a quantitative PCR based melt curve analysis for simple, rapid, and accurate identification of six lepidopteran pests of almonds and pistachios: navel orangeworm, peach twig borer, oriental fruit moth, obliquebanded leafroller, raisin moth, and Indian meal moth. We demonstrate that the dissociation or the “melt” temperature(s) of a 658 bp section of cytochrome c oxidase subunit 1 provides unambiguous species level identification of these six species and is reproducible in field specimens collected following conventional orchard practices. The melt curve’s simplicity allows it to be performed in any basic molecular biology laboratory with a quantitative PCR.

**Abstract:**

Almonds and pistachios are fed upon by a diverse assemblage of lepidopteran insects, several of which are economically important pests. Unfortunately, identification of these pests can be difficult, as specimens are frequently damaged during collection, occur in traps with non-target species, and are morphologically similar up to their third instar. Here, we present a quantitative PCR based melt curve analysis for simple, rapid, and accurate identification of six lepidopteran pests of almonds and pistachios: navel orangeworm (*Amyelois transitella*), peach twig borer (*Anarsia lineatella*), oriental fruit moth (*Grapholita molesta)*, obliquebanded leafroller (*Choristoneura rosaceana)*, raisin moth (*Cadra figulilella*), and Indian meal moth (*Plodia interpunctella*). In this approach, the dissociation (melt) temperature(s) of a 658 bp section of cytochrome c oxidase subunit 1 was determined using quantitative PCR (qPCR). Within these six species, the distribution and the number of melt peak temperatures provide an unambiguous species level identification that is reproducible when unsheared DNA can be extracted. The test is robust across a variety of sampling approaches including insects removed from sticky card traps, museum specimens, and samples that were left in the field for up to 7 days. The melt curve’s simplicity allows it to be performed in any basic molecular biology laboratory with a quantitative PCR.

## 1. Introduction

The almond (*Prunus dulcis*) and the pistachio (*Pistachia vera*) industries are a dynamic part of California agriculture, contributing more than $21 billion to the state’s economy through direct and indirect outputs [1]. Lepidopterans are the primary insect pests in these nut crops [2,3], with the navel orangeworm, *Amyelois transitella* (Walker) (Pyralidae), being arguably the most important pest of the nut hull and meat in both crops [4]. The peach twig borer, *Anarsia lineatella* (Zeller) (Gelechiidae), is most often considered a stone fruit pest [5,6] but was the key almond pest before the arrival of the navel orangeworm and continues to be a primary concern for almond growers [7]. Similarly, oriental fruit moth, *Grapholita molesta* (Busck) (Tortricidae), is not only a stone fruit pest but its larvae can feed on growing almond shoot tips and damage the nut directly [8]. Obliquebanded leafroller, *Choristoneura rosaceana* (Harris) (Tortricidae), is primarily a pest of pistachio rather than almonds, feeding mostly on the leaves, although the larvae occasionally will enter the pistachio nut [9]. Finally, raisin moth, *Cadra figulilella* (Gregson) (Pyralidae) (Gregson), and Indian meal moth, *Plodia interpunctella* (Hübner) (Pyralidae), are post-harvest pests known to feed on almonds drying on the orchard floor as well as in the stored nuts both pre- and post-processing [10].

Most almond and pistachio growers focus pest control efforts against navel orangeworm, with programs focusing on cultural practices such as winter sanitation of unharvested “mummy” nuts [11,12,13], early harvest [14], and growing almond varieties that have a seasonally later or a physically smaller hull split [15]. Mating disruption became an effective and increasingly popular navel orangeworm management tool since the advent of aerosol-based formulations of the pest’s sex pheromone [16,17]. However, even with these practices in place, farm managers are reliant on applications of insecticides during key crop developmental periods such as hull-split. The frequency and the timing of applications are informed by adult monitoring using sex pheromone traps and by counting larvae in mummy nut samples. Heavy pesticide use led to pyrethroid resistance in some populations [18] mandating a search for alternative insecticides of lepidopteran pests [7,19].

**Figure 1:**
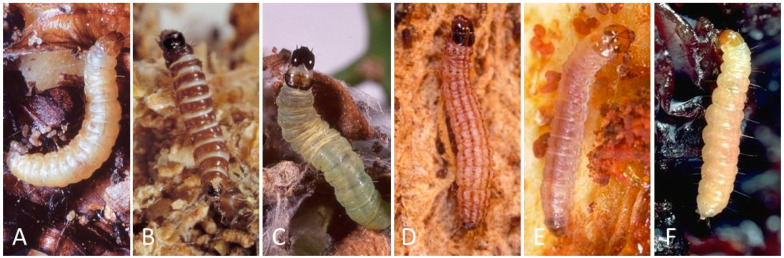
Examples of some of the moth larvae tested, with photos of the fifth instars, which are more easily separated visibly from each other (**A**) navel orangeworm, (**B**) peach twig borer, (**C**) obliquebanded leafroller, (**D**) raisin moth, (**E**) omnivorous leafroller, and (**F**) Indian meal moth. Earlier stages, especially first and second instars, are more difficult to distinguish.

Given the high crop value of almond and pistachio as well as the large number of lepidopteran pest species that are found in orchards, growers are known to apply insecticides after finding any lepidopteran feeding in the nut or on the foliage. Pheromone monitoring can help identify the more numerous moth pest species in the orchard [12,16,20,21,22], but other moth species are frequently captured in the sex pheromone traps and are often difficult to identify due to trap damage or loss of scales [23]. Similarly, larvae found in nuts or leaves are often dead or degraded by the time they are inspected. Even intact live larvae are difficult to identify morphologically in the first thorough the third instars, as these early life stages lack distinctive pigmentation (Figure 1). While a few unknown insects in an orchard are typically inconsequential to crop damage, a large number of pests found in the field can have significant management implications, requiring rapid identification to the species level. Similarly, a small number of unknown larvae found in a shipping container of nuts can result in the refusal of an entire shipment at the cost to the exporter. Considering this, there are a number of economically significant scenarios in which growers, pest control advisors, packhouse employees, or customs agents may encounter moths that they are unable to identify. The identification tools currently available—morphological identification, sequencing of genetic material, and professional identification via government services or private enterprise—are inadequate due to their high cost, lack of accuracy, or time-consuming nature. Consequently, there is a need for an accurate, inexpensive, and rapid identification tool for these lepidopteran pests.

Genetic identification methods targeting the sequence of barcode genes offer an accessible and inexpensive toolbox that is resilient to specimen damage, life stage, and collection technique. These techniques are used broadly to identify insects [24], including some lepidopteran pests [25,26,27]. While a variety of PCR based tools are used for insect identification, the majority of assays employ multiplex PCR. In this approach, a mixture of species-specific primer sets are combined in a single reaction volume [26,28,29,30,31]. These primers are designed to amplify PCR products that vary in length by species and can be rapidly differentiated by visualization on an agarose gel. A distinct drawback of this approach is the need to develop specific primers for each species to be detected. This requirement limits the number of species that can be accommodated by the target gene’s length and genetic variability. Further, introducing additional species increases the chances of primer interactions, which could result in failed PCRs, non-specific amplification, and mis-priming [32,33].

Melt curve analysis is a recently developed diagnostic approach that uses quantitative PCR to indirectly detect small differences in nucleotide sequence between specimens. In this approach, a pair of universal primers are used to amplify a barcode region of the genome; the resulting product is then incrementally heated until the double-stranded DNA dissociates (melts) into two single-stranded molecules. This melting temperature is expected to be species-specific, as it is largely determined by the PCR product’s length and GC content [34]. Though this approach is relatively new, melt curve analysis was successfully used to identify morphologically similar insects in Coleoptera, Lepidoptera, Diptera, and Hemiptera [35,36,37,38,39]. Within animal systems, melt curve analysis most commonly targets mitochondrial genes due to their high copy number, haploid nature, and high mutation rate [40]. The most commonly targeted gene for insect melt curve analysis is cytochrome c oxidase 1 (COI) [35,36,37,39] due to its length, heterogeneity, and the conservation of its sequence and structure across insects [40]. However, the ribosomal intertranscribed spacer (ITS) was used in at least one system [38].

Here, we describe the development of a melt curve analysis for the identification of six lepidopteran pests of almond and pistachio: navel orangeworm, peach twig borer, oriental fruit moth, obliquebanded leafroller, raisin moth, and Indian meal moth. We discuss the usefulness of this method for management of these moth pests in California and its potential as a baseline for the development of diagnostic tools for invasive species.

## 2. Materials and Methods

### 2.1. Insect Specimens

Navel orangeworm, peach twig borer, obliquebanded leafroller, raisin moth, and oriental fruit moth were collected in summer 2017 and 2018 in Fresno County, CA from infested almond and pistachio by hand (immature) or in pheromone baited delta traps (adult). Pheromone trap collected adult specimens were identified to species using morphological features. Ambiguous or damaged adults that could not be readily identified were excluded from analysis. Navel orangeworm, peach twig borer, Indian meal moth, and raisin moth (adults and larvae) were also obtained from insect colonies at the University of California’s Kearney Agricultural Research and Extension Center (KARE) (Parlier, CA), the USDA-ARS, San Joaquin Valley Agricultural Sciences Center (SJVASC) (Parlier, CA), and the Jordan Agricultural Research Center (JARC) at California State University, Fresno (Fresno, CA). Dried and pinned specimens (adult) and voucher larval specimens in 95% EtOH from the Daane lab insect collection were used as needed for peach twig borer, oriental fruit moth, and obliquebanded leafroller. Genomic DNA was extracted from ≤30 mg of whole tissue using the Omega Bio-tek E.N.Z.A. Tissue DNA kit (Omega Bio-tek, Inc., Norcross, GA, USA) with at least 27 specimens processed for each species.

### 2.2. DNA Barcoding

This melt curve analysis targets the 658 bp Folmer Region of the mitochondrial cytochrome c oxidase 1 (COI) gene. The Folmer region encompasses the terminal 650 bp at the 5′ end of the cytochrome oxidase gene. This section of COI was initially identified as the target region for genetic barcoding in the early 1990s because it is flanked by conserved sequences that allow for the design of universal primers, allowing for simplified coordination of DNA sequencing efforts among labs [40,41]. In this analysis, the universal insect primer sets LepF1 (5′-ATT CAA CCA ATC ATA AAG ATA TTG G-3′) and LepR1 (5′-TAA ACT TCT GGA TGT CCA AAA AAT CA-3′) were used due to their wide acceptance and ability to effectively amplify the Folmer region in >120 insect families [42]. Amplification and visualization were performed on the CFX-96 quantitative thermocycler (Bio-Rad Life Science, Hercules, CA, USA) using EXPRESS SYBR GreenER qPCR Supermix (Invitrogen, Carlsbad, CA, USA). PCR conditions were a 2 min activation at 50 °C, a 2 min denaturation at 95 °C, followed by 40 cycles of 15 s at 95 °C and 60 s at 47 °C. The melt curve protocol involved an increment of 0.2 °C increase every 5 s from 55–95 °C. Melt profile and peak charts were generated using the CFX Bio-Rad manager software, which plotted the negative derivative of fluorescence vs. temperature (−dF⁄dT vs. T). The software further identified melt peak temperatures. Mean melt peak temperature (T_m_, the temperature of greatest strand dissociation) values were calculated for replicate analysis of each specimen prior to species mean and 95% confidence intervals being calculated.

All melt curve reactions were repeated in duplicate using at least 27 individuals per species, including both larval and adult stages. In addition to colony reared specimens, the melt curve analysis was performed on field collected samples where available, including adults captured in adhesive pheromone traps (all species except Indian meal moth) and larvae collected from unharvested mummy nuts (navel orangeworm and peach twig borer). Preserved collection specimens were used on occasion (obliquebanded leaf roller, peach twig borer, oriental fruit moth, and navel orangeworm).

### 2.3. Field Assessment of Test Accuracy

To assess the robustness of the melt curve assay under field conditions, the protocol was applied to a random sampling of moth species collected from a California tree nut orchard. Non-target moths were collected from an almond orchard in Madera County California in August of 2019 using “Peterson” style kairomone bait traps. Trap liners were removed every week and were stored at −20 °C prior to analysis. A total of 44 moths were randomly selected from the liners for analysis using the protocol outlined above. In order to identify the randomly sampled moths to species, the COI gene was Sanger sequenced (Functional Biosciences, Madison, WI, USA) using LepF1 and LepR1 primers. The COI sequences were trimmed, aligned, and sorted into taxonomic groups on MEGA-X [43]. Taxonomic groups were identified to species using the Identification Engine tool found on the Barcode of Life Database (BOLD) [44] website with a 98% similarity cutoff. In addition to randomly collected moths from the field, melt curve analysis was performed on eight codling moth, *Cydia pomonella* (L.) (Lepidoptera: Tortricidae), larvae which were sourced from colonies at the USDA San Joaquin Valley Agricultural Sciences Center.

## 3. Results

### 3.1. Melt Curve Profiles of Target Pests

**Figure 2:**
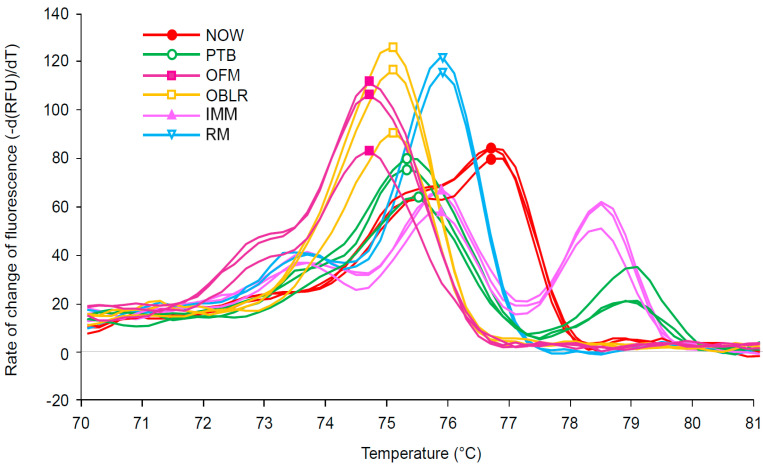
Melt peak chart showing the melt profile for PCR amplicons of the cytochrome oxidase subunit I gene from navel orangeworm (NOW-red), peach twig borer (PTB-green), oriental fruit moth (OFM-purple), obliquebanded leafroller (OBLR-orange), Indian meal moth (IMM-pink), and raisin moth (RM-blue); a symbol was added to the peak point of each melt curve. Only three individuals per species are included in the figure to aid readability.

All six target species produced distinct and unambiguous melt curve profiles, allowing rapid identification to the species level (Figure 2, Table 1). A species’ melt curve profile, when plotted using the negative derivative of fluorescence vs. temperature, is composed of one or more “peaks” which represent dissociation events. Species vary in the number of peaks that are observed and the temperature at which these peaks occur. Three of the target species produced a single melt peak: oriental fruit moth at 74.4 °C, obliquebanded leafroller at 75 °C, and navel orangeworm at 76.6 °C. None of the peaks fell within one another’s 95% confidence interval, suggesting that it is highly unlikely that any of these three species would produce a peak sufficiently divergent from the mean as to cause misidentification.

Three species produced multiphasic melt profiles, i.e., profiles with more than one peak. Two species produced two distinct melt peaks: peach twig borer and raisin moth (Figure 2, Table 1). While the primary peaks of these species fell within 0.4 °C of one another, they were statistically distinct (Table 1). Additionally, the secondary peaks of these species were diagnostic, differing by 5.4 °C. Indian meal moth was unique among the specimens in producing three distinct peaks (Figure 2, Table 1). While peak breadth and height occasionally varied within species, the number of melt peaks and the melt peak temperatures were found to be consistent. 

Melt profiles were found to be consistent within species irrespective of life stage, collection location, and collection method (lab reared, trap collected, insect collection, and field collected larvae).

### 3.2. Field Collected Specimens

**Figure 3:**
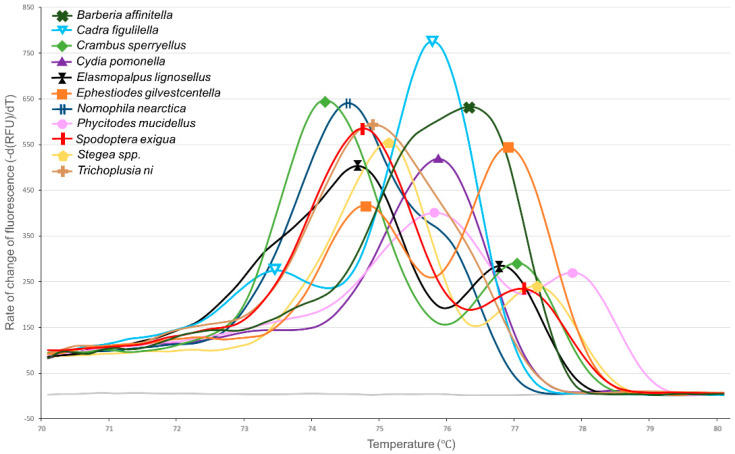
Melt peak chart showing melt profiles for PCR amplicons of the cytochrome oxidase subunit I gene from codling moth and the ten species collected from kairomone traps in almond orchards. Due to the large number of species being represented, only one representative individual is included per species to aid readability.

Of the 44 moths randomly selected from the kairomone trap liners, all successfully amplified COI and produced melt curves. Ten distinct melt curve profiles were identified (Figure 3, Table 2), suggesting the presence of ten distinct species. One of the ten profiles was consistent with the melt pattern of raisin moth, producing a primary peak at 75.8 °C and a secondary peak at 73.6 °C. The remaining nine melt profiles were unique to this work, failing to match any of the six target species previously analyzed. Alignment of COI sequences revealed 10 distinct species clusters among the 44 selected moths, with sequence clusters corresponding to the 10 melt peak profiles. When compared to sequences in BOLD, nine of the ten species clusters could be identified to the species level with a percent identity score > 99.6%. These nine species level identifications included the raisin moth and eight non-target species (Table 2). These species included two occasional orchard pests—the beet armyworm (*Stegea exigua*) and the dusky raisin moth (*Ephestiodes gilvestcentella*). One cluster could not be identified to the species level, sharing 99.65% identity to an unknown member of the genus *Stegea* (family: Crambidae). The sequences did share 96.35% identity with *Stegea eripalis* (Grote), which was below the 98% threshold set for species identification. Codling moth was also found to produce a unique melt curve profile that did not match any of the six target species, producing a single peak at 75.8 °C (Table 2, Figure 3).

One specimen (*Trichoplusia ni*, cabbage looper) had a primary melt peak of 74.8 °C ± N/A (Figure 3), similar to that of oriental fruit moth 74.6 °C ± 0.04 (Figure 2). However, the two species may be easily differentiated by melt peak shape, as oriental fruit moth has a protracted initiation of melting beginning at 72 °C, while cabbage looper does not. That said, it should be noted that the purpose of analyzing field collected specimens was to test the sensitivity of melt curve analysis to false positives and negatives, not to determine the melt profiles of these pests. Given the low sample size of specimens collected per species (≤8), the results in table two have low statistical power and need to be verified with additional samples. Further samples may help elucidate similarities and differences between samples.

## 4. Discussion

A plurality of molecular approaches are employed to identify insects [24] and prove indispensable in systems where species are cryptic [45], difficult to rear [46], or require rapid identification [47]. However, their application is often limited by the need for species specific primers, especially in systems with elevated species diversity or limited genetic diversity between targets. Here, we described the development of a melt curve profile diagnostic tool for the identification of six lepidopteran pests of California tree nuts. The melt curve tool utilized the universal insect primers LepF1 and LepR1 to amplify the 658 bp Folmer region of the mitochondrial COI gene [48], negating the need for species-specific primers. Further, the test proved capable of reliably differentiating the six target species from one another and eleven non-target species.

The developed assays would be particularly useful when collected nut pests cannot be identified in the traditional manner because key morphological features are damaged or specimens are present only in early instars. Under these conditions, identification can be time consuming and can require a high level of expertise. Melt curve analysis is particularly well suited to this scenario considering its short duration (<3 h) and low per sample cost (<USD 3.00 in reagents). While the assay itself is inexpensive, its application may be limited by the relatively high cost of a qPCR instrument. Consequently, diagnostic melt curve assays are likely limited to established molecular biology laboratories, requiring samples be sent to a centralized facility.

It is notable that many diagnostic melt curve analyses target amplicons that are less than 200 bp in length, as melt curve shifts due to single nucleotide differences are more pronounced in a shorter PCR product [49,50]. However, in this work, we demonstrated that the six target species could be readily identified using the melt curve profile of the 658 bp Folmer region of COI (Figure 2). While longer amplicons are less sensitive to individual nucleotide differences, they are more likely to contain multiple GC rich regions, allowing for multiphasic melt curves. Multiphasic curves allow differentiation of species provided at least one region of the amplicon exhibits sufficient differences in GC content between species to shift the dissociation temperature [51]. Considering this, longer amplicons can be intentionally selected for melt curve analysis to increase the number of species that can be differentiated [52]. The benefit of this approach is evident in this work when randomly sampled moths were tested. Two of the kairomone sampled moths produced primary melt peaks at 75.8 °C, which is identical to the primary melt peak of raisin moth. However, these non-target species were easily differentiated from raisin moth by the presence of a non-matching secondary peak (*Phycitodes mucidellus*) or a complete lack of secondary peak (codling moth) (Table 2). These findings are in line with those of Winder [39], where three beetle pests were differentiated using melt curve analysis of a 1135 bp amplicon of COI. Given that the LepF1 and the LepR1 primers used in this test are capable of amplifying the Folmer region across most insect groups [42], they could potentially be used to quickly develop diagnostic melt curve analyses in a wide range of ecosystems/species complexes. There also appears to be significant potential for LepF1 and LepR1 to be used in melt curve analyses outside of the United States, as these primers were also widely used by the Biodiversity Institute of Ontario in their Barcoding of Life Database (BOLD) effort [53], which includes COI sequences from insects collected on every continent. 

A potential pitfall of molecular diagnostics is their reliance on high-quality DNA, which can be difficult to collect from field specimens. Adults captured from pheromone traps may have been exposed to the elements for several days, allowing DNA degradation [54]. Trap captured moths are also typically coated in an adhesive, which interferes with taxonomic identification and introduces a potential source of PCR inhibitors. DNA contamination or damage is particularly challenging to qPCR-based assays such as the melt curve analysis. Low quality DNA can result in nonspecific PCR products or late amplification, both of which can produce incorrect melt curve profiles, leading to a false positive or negative. Alternatively, heavily damaged DNA may completely fail to amplify, rendering the insect impossible to identify. Despite these challenges, this melt curve analysis was capable of quickly and accurately identifying 1- to 3-week-old specimens collected in sticky card traps. For example, all of the kairomone sampled moths from Madera County were selected from 7-day-old trap liners. Despite this, COI successfully amplified on the first attempt in all 44 samples and produced melt curve profiles that corresponded to their species. Orchard moth traps are typically checked once every week, suggesting that the melt curve analysis as designed would be robust to the most common orchard monitoring practices.

Another concern that should be addressed is the possibility of false positives. False positives occur in melt curve analysis when the melt peaks of a non-target species produce a reasonable facsimile of the target species’ melt curve profile. False positives can be difficult to predict, as melt curves are determined by complex interactions between nucleotides, and dissimilar sequences can occasionally produce similar melt peak profiles. Indeed, it is unlikely that a non-target species and a pest would have nearly identical COI sequences, as the congeneric diversity at COI is estimated at 6.6% [47] and the confamilial diversity at 11.3% [46] in Lepidoptera. The only reliable method for ruling out false positives is extensive testing of species that cohabitate the ecosystem. This is not entirely feasible in this case, as production orchards are home to a diverse range of pest and non-pest moths, many of which do not have existing pheromones for sampling purposes. Consequently, instead of taking a targeted approach, we utilized a kairomone trap designed to attract any moths that oviposit on tree nuts. Using these randomly sampled moths, our melt curve test failed to identify any identical melt curves among the six target and the eleven non-target species that were analyzed. These specimens include the most common insect pests found in tree nut orchards (the six target species), as well as the occasional pests: codling moth, beet armyworm, and dusky raisin moth. Considering this, the Lepidopteran species most commonly collected in California tree nut orchards would be unlikely to produce a false positive. However, the kairomone trap collected moths from Madera County included several species that are not associated with orchards. These samples included agricultural pests such as the sodworm *Crambus sperryellus* (Klots), brassica pest *Trichoplusia ni* (Hübner), row crop pest *Elasmopalpus lignosellus* (Zeller), and several species that are not known agricultural pests. This suggests that orchards may be home to a diversity of species that are either transient or feeding on neighboring crops, drive row cover crops, or weeds. Considering the diversity of lepidopteran species present in tree nut orchards, it may be impossible to develop a molecular diagnostic test that is 100% robust to false positives. To offset this challenge, it would be beneficial for laboratory technicians to collect basic morphological data such as moth size and color to rule out obvious false positives.

Another potential challenge to this assay is false negatives due to COI haplotypes within the target species, or due to the presence. Intraspecific genetic diversity in the COI gene may be sufficient to produce melt curves that deviate from the expected patterns discovered here, resulting in a false negative. High resolution melt curve is sufficiently sensitive to detect minor deviations in sequence. Most notably, Swisher et al. [37] were capable of differentiating three haplotypes of *Bactericera cockerelli*, two of which only differed by a single nucleotide within the 500 bp amplicon. The Californian populations of the six species targeted by our assay were not subject to widespread COI sequencing. Consequently, the haplotypic diversity of these populations is currently unknown. The moths tested in the development of this assay did not exhibit any intraspecies diversity in terms of peak number, while deviations in peak temperature were uncommon and never exceeded 0.2 °C. This suggests that either the tested specimens shared a haplotype within species or that any haplotypes present were not sufficiently divergent to alter the species’ melt profile. However, without extensive testing of field populations from across the pest’s geographic range, it is impossible to rule out the possibility of melt shifting haplotypes. If melt shifting haplotypes were discovered in a target species, the new curve could be added to the melt curve assay as an alternative profile for the pest upon verifying the species identity via COI sequencing. 

## 5. Conclusions

This work reports on a novel methodology for tree nut pest identification. The system used was a group of lepidopterous pests that attach nut crops in the western USA and which are commonly found in young larval stages or poor shape as adults in pheromone traps, both of which can leave individuals difficult to identify using conventional approaches. We present a quantitative PCR based melt curve analysis for simple, rapid, and accurate identification of six lepidopteran pests of almonds and pistachios. We show that the dissociation or the “melt” temperature(s) of a section of cytochrome c oxidase subunit 1 can be determined using quantitative PCR to provide species identification and is reproducible when unsheared DNA can be extracted. The melt curve’s simplicity allows it to be performed in any basic molecular biology laboratory with a quantitative PCR and could be easily expanded to include additional pests of economic interest. We discuss some potential concerns with this methodology, such as the collection of high-quality DNA and DNA contamination or damage that can be challenging to qPCR-based assays such as the melt curve analysis described here. Another concern is false positives that can be difficult to predict, but we argue that it is unlikely that a non-target species would have nearly identical melt curve patterns. Similarly, we explore the possibility of false-negatives due to haplotypic diversity and note that there is currently no evidence of melt curve shifting haplotypes within the target species. We suggest that melt curve analysis may be used for species identification, especially where costs or time are limiting, and suggest further development of melt curve identification for different systems will better test this approach as an alternative or addition to PCR analyses. 

## Figures and Tables

**Table 1:** Melt peak profile for examined pests including estimated melt peak temperature (T_m_) for both primary and secondary peaks with 95% confidence interval.

Species	Primary Melt Peak T_m_ (°C)	Secondary Melt Peak(s) T_m_ (°C)	Individuals Tested (N)
Navel orangeworm	76.6 ± 0.04		36
Peach twig borer	75.4 ± 0.04	79.0 ± 0.04	27
Oriental fruit moth	74.6 ± 0.04	-	32
Obliquebanded leafroller	75.0 ± 0.02	-	36
Indian meal moth	78.4 ± 0.04	75.8 °C ± 0.04, 73.6 ± 0.07	34
Raisin moth	75.8 ± 0.05	73.6 ± 0.07	34

**Table 2:** Melt peak profile for codling moth and the ten species collected from kairomone traps in almond orchards. Data include estimated melt peak temperature (T) for both primary and secondary peaks with 95% confidence interval and the number of individuals collected for each species.

Species (and Common Name)	Family	Primary Melt Peak T (°C)	Secondary Melt Peak T (°C)	Individuals Collected (N)
*Cadra figulilella* (raisin moth)	Pyralidae	75.8 ± 0.10	73.6 ± 0.07	16
*Cydia pomonella* (Codling moth)	Tortricidae	75.8 ± 0.06	-	8
*Spodoptera exigua* (beet armyworm)	Noctuidae	74.6 ± 0.08	77.0 ± 0.12	8
*Barberia affinitella* (N/A)	Pyralidae	76.2 ± 0.00	-	7
*Ephestiodes gilvescentella* (dusky raisin moth)	Pyralidae	76.6 ± 0.23	74.8 ± 0.25	4
*Nomophila nearctica* (Lucerne moth)	Crambidae	74.4 ± 0.00	-	3
*Stegea spp.* (N/A)	Crambidae	75.0 ± 0.00	77.2 ± 0.00	2
*Crambus sperryellus* (Sperry’s lawn moth)	Crambidae	74.2 ± N/A	77.0 ± N/A	1
*Elasmopalpus lignosellus* (lesser cornstalk borer)	Pyralidae	74.6 ± N/A	76.8 ± N/A	1
*Phycitodes mucidellus* (N/A)	Pyralidae	75.8 ± N/A	77.8 ± N/A	1
*Trichoplusia ni* (cabbage looper)	Noctuidae	74.8 ± N/A	-	1

## Data Availability

Data are available upon request.
